# Dental public health – principles and practice

**DOI:** 10.1038/s41415-026-0188-4

**Published:** 2026-11-13

**Authors:** Ivor G. Chestnutt

**Affiliations:** British Association for the Study of Community Dentistry, UK; Dental Public Health, Cardiff University School of Dentistry, Cardiff, United Kingdom

## Abstract

With origins in the early 20^th^ century and closely allied to community-based dentistry, local authorities, education and generic public health, dental public health is a recognised clinical dental specialty that deals with prevention of oral disease and facilitates the adoption of effective and efficient dental care and interfaces with institutions and agencies outside the health sector. This manuscript describes the components of dental public health, the public health workforce, the challenges currently faced and the opportunities ahead.

## Key points

- Dental public health is the discipline that deals with the improvement of oral health and delivery of dental care from a population rather than individual patient perspective.
- Those involved in public health work across a range of sectors including local authorities, education and social work to address the major determinants of oral diseases and address health inequalities.
- Recent changes to how public health is delivered and how the National Health Service in England is structured have impacted on how the dental public health function is organised and delivered.

## What is dental public health?

Dental public health is defined as: ‘The science and art of preventing oral disease, promoting oral health and improving the quality of life through the organised efforts of society'.

The discipline has its origins in the early 20^th^ century and Gelbier's comprehensive review of the development of dental public health has recently been published in this journal.^1^ The specialty of dental public health as currently understood dates from the establishment of a Specialty Advisory Committee in April 1991. It is now one of the thirteen specialties recognised by the General Dental Council (GDC).

## The components of dental public health

**Fig. 1:**
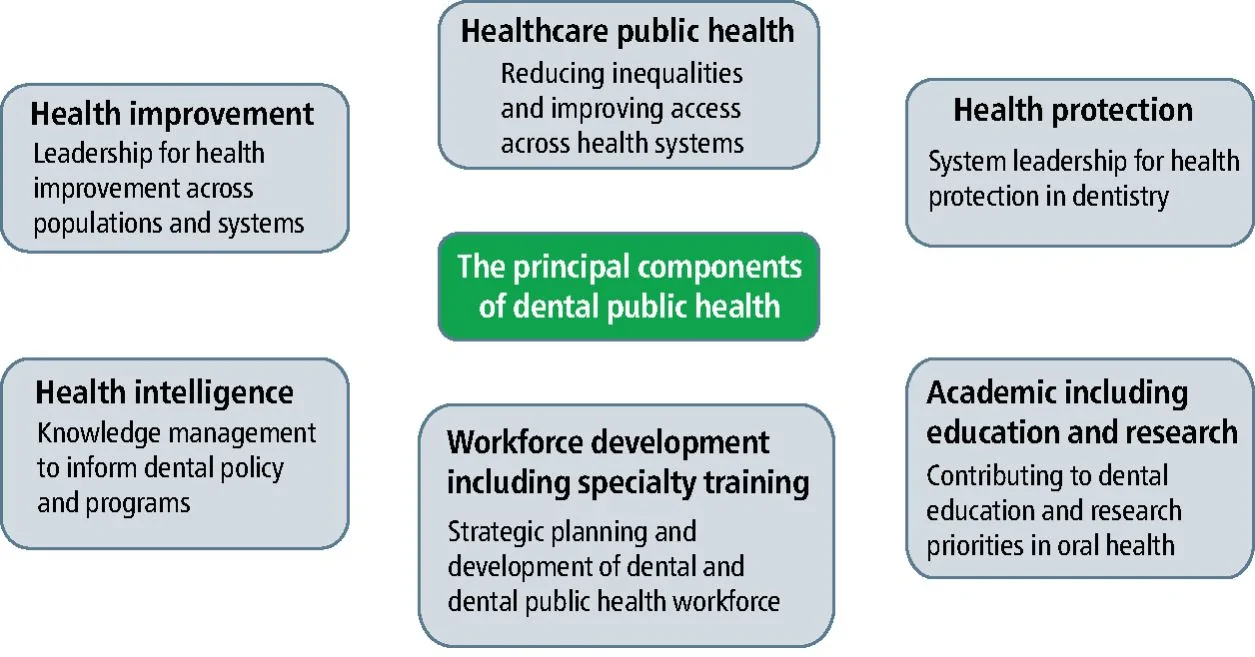
The principal components of dental public health

The work of consultants, specialists and practitioners in dental public health is spread across six domains (Fig. 1).^2^

### Health improvement

Perhaps the aspect that is most closely associated with the discipline, health improvement is central to the dental public health mission. While all members of the dental team have prevention in their remit, dental public health practitioners have a particular role in working across sectors both within health and more widely to lobby for upstream actions to modify environments and encourage lifestyle and life-circumstance change. This is achieved via collaboration with colleagues in general public health and in local authorities.

Providing leadership in the development of local, regional and national oral health improvement strategies, their implementation and evaluation is key to the discipline.

### Healthcare public health

Healthcare public health centres on reducing inequalities and improving quality and access across healthcare systems. Working with National Health Service (NHS) and local authority partners, the objectives of healthcare public health are to maximise the safety, value for money, efficiency, equity and effectiveness of clinical dental services. Working with other clinical disciplines, the coordination of integrated care pathways, especially in coordinating between primary and secondary care and the development of managed clinical networks are important.

### Health protection

Health protection forms a central part of the generic public health function. Those working in dental public health have a role to play in providing dental advice in the investigation and management of incidents in dental practices. Advice to statutory bodies involved in the inspection and regulation of infection prevention and control in clinical settings is part of the role.

Occasionally dental public health practitioners become involved in the management of situations where a member of the dental team is diagnosed with a blood borne virus or in scenarios where infection control procedures in a practice have been called into question.

### Health intelligence

Collating and analysing data to inform dental policies and programmes are a further important function of dental public health. Working in collaboration with community and public dental health services, the routine conduct of epidemiological surveys of school children and other specific population groups such as care and nursing home residents is important in providing a picture to government and commissioners on the changing patterns and distribution of dental and oral disease.

What we know about the changes in oral health of adults, the impact of social and economic deprivation and the impact of interventions such as the introduction of fluoride toothpaste and the availability of fluoride in the public water supply are all derived from dental epidemiological surveys.

The UK-wide decennial epidemiological surveys have fallen by the wayside because of the differing needs of the devolved administrations and the costs and administrative burden involved with such exercises. The system is therefore now reliant on country-specific surveys.

The analysis of dental service information and the conduct of health needs assessments are used to support planning, procurement, commissioning, monitoring and service evaluation. This is important in arguing the case for investment in dentistry as opposed to the many other calls on the healthcare public purse.

### Workforce development including specialty training

Understanding dental workforce capacity and the possibilities of skill-mix in the delivery of care are important attributes informing future workforce needs and the deployment of resources at both national and local levels. Future workforce planning is notoriously difficult. The population-wide view of dental needs and service availability in workforce planning is provided by those with public health skills.

Senior practitioners in dental public health are involved in training the next generation of dental public health practitioners. The standard training is four years in duration, including the completion of a master's degree in public health or dental public health. This degree is central to gaining knowledge to a depth and breadth not covered in the standard undergraduate curriculum and to enable those from a dental background to function in the multidisciplinary setting that constitutes modern public health teams.

On completion of training, candidates must pass the Dental Specialty Fellowship Examination. The learning outcomes and competencies to be achieved by completion of training are described by the GDC's postgraduate specialty training curriculum.^3^ The subsequent award of a Certificate of Completion of Specialty Training by the relevant postgraduate authority is required for entry to the GDC's specialist list.^4^

### Academia including education and research

A significant proportion of dental public health consultants and specialists are employed in academia and their work involves the generation of evidence on health, health risks and health services and promoting the use of evidence-based practice in dental services.

Knowledge and expertise from other academic disciplines such as:

- Sociology (to understand the development, structure and function of human societies)
- Psychology (important in understanding and facilitating behaviour change)
- Health economics (in explaining the need for, demand and supply of resources and healthcare services)
- Medical statistics (in understanding the inferences that can reasonably be drawn from data).

These are all areas with which academics in dental public health interface on a regular basis.

A large number of learning outcomes expected of graduating dental professionals have their basis in dental public health and academics in the specialty are responsible for teaching these to undergraduate students of dentistry, dental hygiene and dental therapy.

## The dental public health workforce

The 2024 GDC annual report showed that there were 83 dentists registered as a specialist in dental public health, down from 98 in 2020 and 126 in 2006, the year in which the number of dentists so registered was highest.^5^^,^^6^

The practice of dental public health is of course not limited to those registered as specialists and many members of the dental team work in areas that are central to what we do. Members of the community and public dental health services are key to the collection of dental epidemiological data and to the implementation of national oral health improvement programmes such as Designed to Smile^7^ and Childsmile.^8^ The latter operates in Scotland and is currently celebrating its 20^th^ anniversary. Childsmile aims to improve children's oral health, reduce inequalities in dental health, and ensure that every child has access to dental services across Scotland. Designed to Smile is a somewhat similar programme in Wales which delivers daily supervised toothbrushing in nurseries and schools, provides ‘home packs' containing toothbrushes and toothpaste and twice-yearly fluoride varnish application to children resident in disadvantaged communities.

The current English Government's supervised toothbrushing programme will be dependent on members of oral health promotion teams and community dental services for its delivery.

Oral health education teams play an important role in delivering health improvement messages in local settings and should be closely aligned with their local dental public health network.

### Employers

As noted above, consultants and specialists in dental public health work either in academia employed by universities, in the national health service or for a few, in the defence dental services.

Academics usually have an honorary contract with a local NHS body and many whose primary employer is the NHS have honorary status with a local higher education institution, primarily to enable them to undertake teaching duties.

In Scotland and Wales, NHS consultants and specialists are usually employed by Public Health Scotland and Public Health Wales. This enables integration with colleagues working in generic public health and has for the past decade provided stability to the NHS public health function in these countries.

In England the situation has in recent years been much more unstable, largely due to the many reorganisations of the NHS structure and public health function. Before 2012, dental public health practitioners were largely employed by either primary care trusts or strategic health authorities. The Lansley reforms saw local authorities become responsible for commissioning public health services, including oral health improvement.^9^ The commissioning of dental services became the responsibility of NHS England. Dental public health staff then found themselves dispersed across, local authorities, NHS England and Public Heath England.

These changes fragmented the dental public health workforce in England and saw many in dental public health separated from their generic public health counterparts.

These reorganisations led to reduced coordination and weakened strategic oversight, and the previously existing lines of accountability and influence were disrupted. Many dental public health professionals left the system due to uncertainty or lack of role. The decentralisation led to geographic variation in access to public health services and roles.

More recent and further disruption affecting the system arose from the abolition of Public Health England (in the midst of the COVID-19 crisis) and then the decision of the Starmer government to disband NHS England. This has severed to further destabilise the dental public health base in England.

## British Association for the Study of Community Dentistry

The official association for those involved in dental public health practice in any capacity is the British Association for the Study of Community Dentistry (BASCD). Now over 50 years old, the Association exists to provide a professional home for all those whose work relates to community dentistry and public health.^1^

The association is a vehicle for sharing experience and professional support for those working in a specialty where they can easily become professionally isolated. Via annual conferences and webinars, BASCD provides discipline-specific continuing professional development. Membership is open to anyone with an interest in community dentistry and dental public health.

## Issues currently facing dental public health

Dental public health has not been immune to the challenges faced by the NHS and NHS dental services in general. The repeated reorganisations of the system just described has disintegrated the pathways for advice and action. Coupled with the 35% reduction in registered specialists over the past two decades, there are questions around the availability of those with the skills and knowledge to fulfil the function. It is crucially important that opportunities are created for those completing the specialty training programmes – there are 16 trainees currently in the system.

Decisions such as that by NHS England not to provide honorary contracts to academics in England has no doubt deprived the NHS of valuable expertise.

## The opportunities ahead

The ten-year plan for England majors on a transition from treatment to prevention and designing oral health strategies that meet with local needs and resources will be important. Only dental public health practitioners have the wherewithal to link oral health into wider health promoting activities. Knowing what to implement, where to implement it and the evaluation of its success is reliant on good quality data that can be derived from dedicated epidemiological data or by linking to other generic public surveys and questionnaires. Dental public health expertise is central to the intelligence needed.

Addressing oral health inequalities is an ever-present challenge and dental public health is key to that mission. Supporting programmes such as the supervised toothbrushing scheme in England, mirroring the success of Designed to Smile^7^ and Childsmile^8^ will be important.

The difficulties encountered by NHS General Dental Services (GDS) have been discussed at length over the past two decades in the pages of this journal.^10^^,^^11^^,^^12^^,^^13^^,^^14^^,^^15^ Contract reform is progressing to different degrees and in a variety of ways across the UK. Whether universal provision of NHS GDS services in the format that they have previously been known will survive is a matter for debate.

However, if the concept of a state-funded dental service that at least provides for those in greatest need is to be preserved, then a functioning dental public health resource will be essential to meet that aspiration.
